# A diketopiperazine factor from *Rheinheimera aquimaris* QSI02 exhibits anti-quorum sensing activity

**DOI:** 10.1038/srep39637

**Published:** 2016-12-21

**Authors:** Shiwei Sun, Xiaoyun Dai, Jiao Sun, Xiangguo Bu, Caihong Weng, Hui Li, Hu Zhu

**Affiliations:** 1 Center for Bioengineering and Biotechnology, China University of Petroleum (East China), Qingdao 266580, P. R. China

## Abstract

An ethyl acetate (EtOAc) extract isolated from the marine bacterium, *Rheinheimera aquimaris* QSI02, was found to exhibit anti-quorum sensing (anti-QS) activity. A subsequent bioassay-guided isolation protocol led to the detection of an active diketopiperazine factor, cyclo(Trp-Ser). Biosensor assay data showed that the minimum inhibitory concentration (MIC) of cyclo(Trp-Ser) ranged from 3.2 mg/ml to 6.4 mg/m for several microorganisms, including *Escherichia coli, Chromobacterium violaceum* CV026, *Pseudomonas aeruginosa* PA01, *Staphylococcus aureus,* and *Candida albicans*. Additionally, sub-MICs of cyclo(Trp-Ser) decreased the QS-regulated violacein production in *C. violaceum* CV026 by 67%. Furthermore, cyclo(Trp-Ser) can decrease QS-regulated pyocyanin production, elastase activity and biofilm formation in *P. aeruginosa* PA01 by 65%, 40% and 59.9%, respectively. Molecular docking results revealed that cyclo(Trp-Ser) binds to CviR receptor more rigidly than C_6_HSL with lower docking energy −8.68 kcal/mol, while with higher binding energy of −8.40 kcal/mol than 3-oxo-C_12_HSL in LasR receptor. Molecular dynamics simulation suggested that cyclo(Trp-Ser) is more easy to bind to CviR receptor than natural signaling molecule, but opposite in LasR receptor. These results suggest that cyclo(Trp-Ser) can be used as a potential inhibitor to control QS systems of *C. violaceum* and *P. aeruginosa* and provide increased the understanding of molecular mechanism that influences QS-regulated behaviors.

The discovery of antibiotics in the early 20^th^ century provided effective control of a large number of life-threatening infections^1^. However, there has recently been an increase in bacterial multi-drug resistance. Due to this observed increase in microbial resistance, antibiotic treatments are becoming more challenging to produce and clinically implement. The documented antibiotic resistance highlights the need for the development of different target processes in the pathogen. One such target system is quorum sensing (QS)^2^,^3^.

QS consists of cell-to-cell communication that depends on the production of, and response to, small diffusible molecules called autoinducers. The most widely studied autoinducers are *N*-acylhomoserine lactones (AHLs) in gram-negative bacteria and peptides in gram-positive bacteria. Additionally, autoinducer-2 (AI-2) is used for interspecies communication between gram-negative and gram-positive bacteria^4^. Research into QS has yielded an increasing number of small chemical compounds that have been shown to act as signal molecules^5^,^6^. Diketopiperazines (DKPs), the smallest cyclic peptides, are commonly isolated from various natural resources such as fungus, lichen, higher marine organisms and gram-negative bacteria^7^,^8^,^9^ and represent a new class of QS signal molecules^7^. Importantly, DKPs are recognized as a group of QS inhibitors that influence cell-to-cell signaling^10^.

In bacteria, QS is the interaction between an autoinducer molecule and its companion acceptor domain. This type of interaction results in the modulation of bacteria to optimize survival. The molecular regulation is in accordance with the density of the population^11^,^12^,^13^. A QS system associated with light production was first discovered in the marine bacterium *Vibrio fischeri*^14^. Bioluminescence QS constitutes a gene regulation system. It involves multiple steps: (1) synthesis of diffusible chemical signals, (2) secretion of the new molecule to the environment, and (3) uptake of the molecules. In general, when the autoinducer concentration reaches a critical threshold, the QS system is activated to express genes associated with that pathway^15^,^16^,^17^. Examples of QS-activated functions include, but are not limited to, the organization of light-emitting reactions (bioluminescence)^18^, the formation of biofilms^19^, the production of antibiotics^20^, spore formation^21^ or the expression of virulence factors^22^,^23^. These pathways, under the control of QS, provide alternative targets for second-generation antibiotics. For example, the biofilm produced by *P. aeruginosa* consists of a highly complex matrix formed on the surface of the bacteria and is regulated by multiple genes^24^. Cyclo(Phe-Pro) can enhance the expression of the protein OmpU, which induces resistance to biofilm formation, attachment to host cells in symbiotic relationships and adhesion^25^. QS-associated inhibition provides an attractive and novel way to treat bacterial infections without accelerating multi-drug resistance. In recent years, natural resources from plants and microorganisms have received attention as sources of bacterial QS inhibitors^26^. Known compounds that have been demonstrated to inhibit the bacterial QS system include *p*-coumaric acid from garlic extract^27^, methyl eugenol from *Cuminum cyminum*^28^, baicalein from *Scutellaria baicalensis*^29^, curcumin from *Curcuma longa*^30^, maniwamycins from *Streptomyces* sp.^31^, unidentified secondary metabolites from marine *Streptomyces*^32^, furanosesterterpenes from the marine sponge *Ircinia felix*^33^, sesqui- and triterpenoids from the liverwort *Lepidozia chordulifera*^34^, and unidentified components from edible mushrooms^35^,^36^.

Marine-derived microorganisms are widely recognized as a significant chemical resource, producing various levels of active secondary metabolites^37^. The data presented here demonstrate extraction and characterization of a compound from *Rheinheimera aquimaris* isolated from a dredge of the Yellow Sea, Qingdao^38^. *R. aquimaris* was first isolated in 2007 from seawater of the East Sea in Korea^39^. At that time, it was identified as a novel species of the genus *Rheinheimera*. To date, there have been few reports on its secondary metabolites and potential biological activities, except for the production of halotolerant amylase^40^. In this study, the use of a bioassay-guided method identified a DKP compound that will be discussed. The compound was characterized as cyclo(Trp-Ser) by NMR and MS spectra. The effect of cyclo(Trp-Ser) on both *C. violaceum* QS-regulated violacein production and *P. aeruginosa* QS-regulated virulence factor production (pyocyanin and elastase) was studied. Additionally, biofilm formation was also assayed. To understand the interaction between the QS inhibitor and receptor proteins, molecular docking and molecular dynamics simulation studies were conducted.

## Results

### Isolation and structure elucidation of QS inhibitors from *R. aquimaris* QSI02

**Figure 1:**
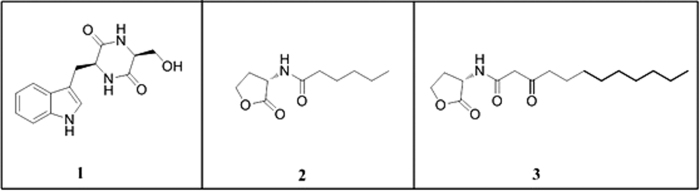
The chemical structure of compound cyclo(Trp-Ser) (1); C_6_HSL (2); 3-oxo-C_12_HSL (3).

The anti-QS activity of the EtOAc extracted from *R. aquimaris* QSI02 was initially tested by treatment of the *C. violaceum* CV026 biosensor system with the extract, as described in the Materials and Methods section. This exposure inhibited violacein production without showing any antibacterial effect, indicating anti-QS activity. Following extraction, 13.8 g EtOAc extract was isolated from 10 L of culture (see the Materials and Methods for details). The compound was further purified by bioassay-guided fractionation using repeated silica gel column purification, Sephadex LH-20, and semi-preparative HPLC. One DKP compound was isolated from the extract. This compound was identified as cyclo(Trp-Ser) (Fig. 1) by ESIMS and NMR analyses and by comparison with literature^41^. The ^1^H-NMR, ^13^C-NMR and MS spectra are shown in Supplementary Figures 1–3, respectively.

### Antimicrobial activity of cyclo(Trp-Ser)

To evaluate the efficacy of cyclo(Trp-Ser) as an antimicrobial agent, *C. violaceum* CV026 and *P. aeruginosa* PA01 were exposed to the compound, and MIC values and growth curve data were collected. The MIC data (see Supplementary Table 1) indicate that gram-negative cells have an elevated MIC compared to gram-positive cells. The gram-negative MICs are as follows: *Escherichia coli*, 6.4 mg/ml; *C. violaceum* CV026, 3.2 mg/ml; and *P. aeruginosa* PA01, 6.4 mg/ml. The gram-positive MICs are as follows: *S. aureus*, 3.2 mg/ml and *Candida albicans* 00147, 6.4 mg/ml.

**Figure 2:**
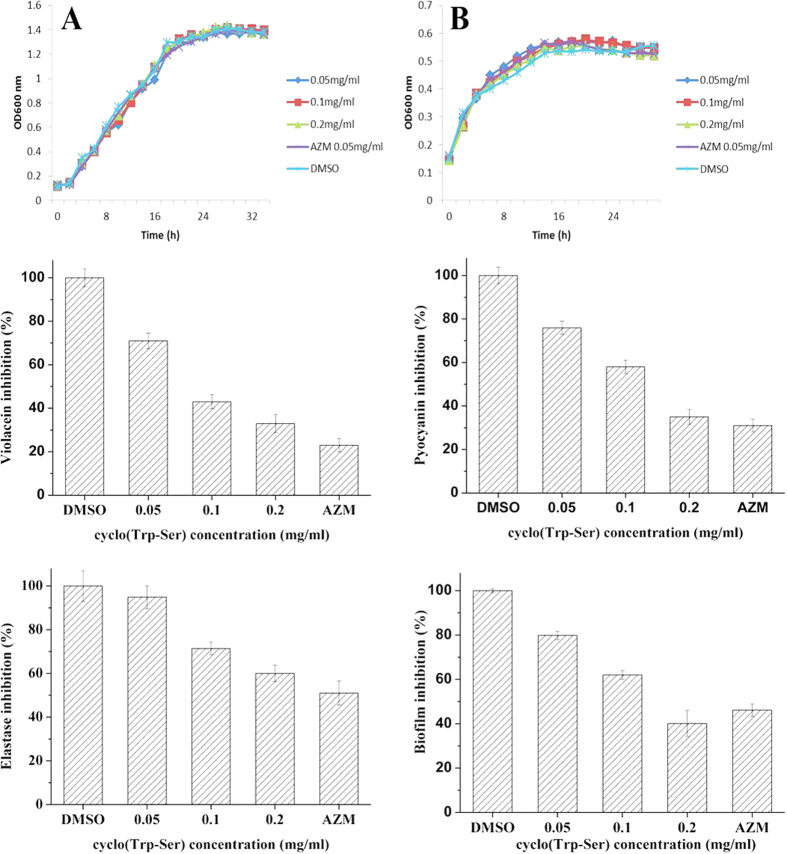
Biological activity of cyclo(Trp-Ser). Growth of *P. aeruginosa* PA01 (**A**), *C. violaceum* CV026 (**B**) in the presence of cyclo(Trp-Ser); The effect of cyclo(Trp-Ser) on violacein production (**C**) in *C. violaceum* CV026; The effect of cyclo(Trp-Ser) on pyocyanin production (**D**), elastase activity (**E**), and biofilm inhibition (**F**) in *P. aeruginosa* PA01. Values are presented as mean ± SD, n = 3. AZM, azithromycin (0.05 mg/ml) and DMSO (1% v/v) were used as positive and negative control respectively.

The growth curves of 0.05, 0.1, and 0.2 mg/ml cyclo(Trp-Ser) and 0.05 mg/ml for the positive control compound, azithromycin (AZM), which has been shown to inhibit the production of several QS-regulated virulence factors of *P. aeruginosa*^42^,^43^,^44^,^45^, are shown in Fig. 2A and B. Neither of these compounds showed a remarkable difference compared with DMSO-treated bacterial cultures. The results indicated that cyclo(Trp-Ser) concentrations lower than 0.2 mg/ml have no effect on the growth of *C. violaceum* CV026 or *P. aeruginosa* PA01.

### Inhibition of QS-regulated violacein production in *C. violaceum*

Violacein production induced by C_6_HSL in *C. violaceum* CV026 is a QS-mediated phenotype. Anti-QS activity was further confirmed by testing for inhibition of violacein production in *C. violaceum* CV026 using 1/32 MIC of cyclo(Trp-Ser). Spectrophotometrical analysis indicated that all tested concentrations of cyclo(Trp-Ser) inhibited violacein production. This inhibitory effect was found to be dependent on the sub-MIC range of 0.05–0.2 mg/ml. At a concentration of 0.2 mg/ml, cyclo(Trp-Ser) inhibited violacein production by up to 67% compared to the control (Fig. 2C).

### Inhibitory effects of cyclo(Trp-Ser) on virulence factor production

Sub-MIC levels of cyclo(Trp-Ser) were used to determine if known QS-dependent pathway components, pyocyanin production and elastase activity are altered upon treatment. The pyocyanin concentration was measured by a quantitative chemical assay, and the results indicate that 0.2 mg/ml cyclo(Trp-Ser) induced remarkable inhibition of pyocyanin production. A 65% decrease with cyclo(Trp-Ser) and a 69% decrease in the presence of the positive control compound, AZM, was observed compared with DMSO (Fig. 2D). Elastase activity was assessed using an elastin-Congo red assay. As shown in Fig. 2E, all tested sub-MIC concentrations of cyclo(Trp-Ser) decreased the elastase activity of *P. aeruginosa* PA01. The most effective concentration was 0.2 mg/ml cyclo(Trp-Ser), which reduced elastase activity by 40%; the positive control induced a 49% inhibition.

### Inhibitory effects of cyclo(Trp-Ser) on biofilm formation

A crystal violet assay was performed, as described in the Materials and Methods section, to determine the effects of cyclo(Trp-Ser) on *P. aeruginosa* PA01 biofilm formation. The results showed that sub-MIC concentrations of cyclo(Trp-Ser) can effectively reduce biofilm formation. Importantly, a 0.2 mg/ml dose of cyclo(Trp-Ser) resulted in a 59.9% reduction of biofilm formation in *P. aeruginosa* PA01. The 0.2 mg/ml concentration performed better than the positive control, which produced only a 53.9% reduction (Fig. 2F).

**Figure 3:**
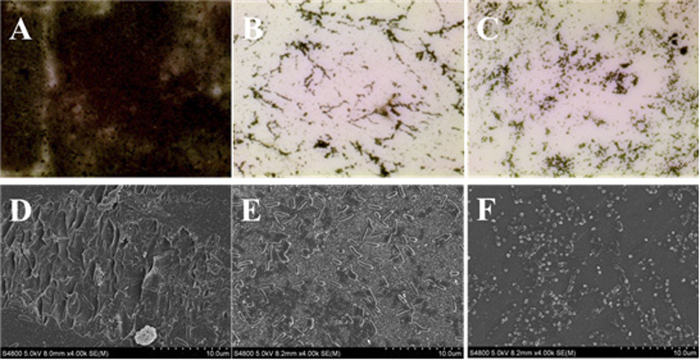
Optical micrographs (**A–C**), (100x) and scanning electron microscope images (**D–F**), (4000x) of *P. aeruginosa* PA01 biofilms after 3 days incubation. (**A,D**) A negative control with DMSO; (**B,E**) 0.2 mg/ml cyclo(Trp-Ser); (**C,F**) a positive control with AZM.

Visual confirmation of the effect of cyclo(Trp-Ser) on biofilm formation was obtained using an optical microscope and a scanning electron microscope (SEM). The optical microscopy analysis after silver staining showed a piece of film-like black floccule on the cover glass in the negative control, but only thin dark specks following treatment with 0.2 mg/ml cyclo(Trp-Ser) or 0.05 mg/ml AZM (Fig. 3A–C). Additionally, a decrease in the amount of *P. aeruginosa* was observed after treatment with either 0.2 mg/ml cyclo(Trp-Ser) or AZM. Only small clusters of individual cells remained attached, unlike the negative control, in the SEM images (Fig. 3D–F).

### Molecular docking analysis

To determine the possibility of binding interactions between the cyclo(Trp-Ser) and QS receptors, docking studies were performed. The crystal structures of QS signal receptor CviR (PDB ID, 3QP1) from *C. violaceum* and LasR (PDB ID, 2UV0) from *P. aeruginosa* were used for the docking calculations. The natural ligands were docked back into the relevant proteins as a control and to establish a credible docking method. The control docking of C_6_HSL into CviR and 3-oxo-C_12_HSL into LasR each resulted in interactions that were highly consistent with those reported in the X-ray structures^46^,^47^ (see Supplementary Figures 4 and 5).

**Figure 4:**
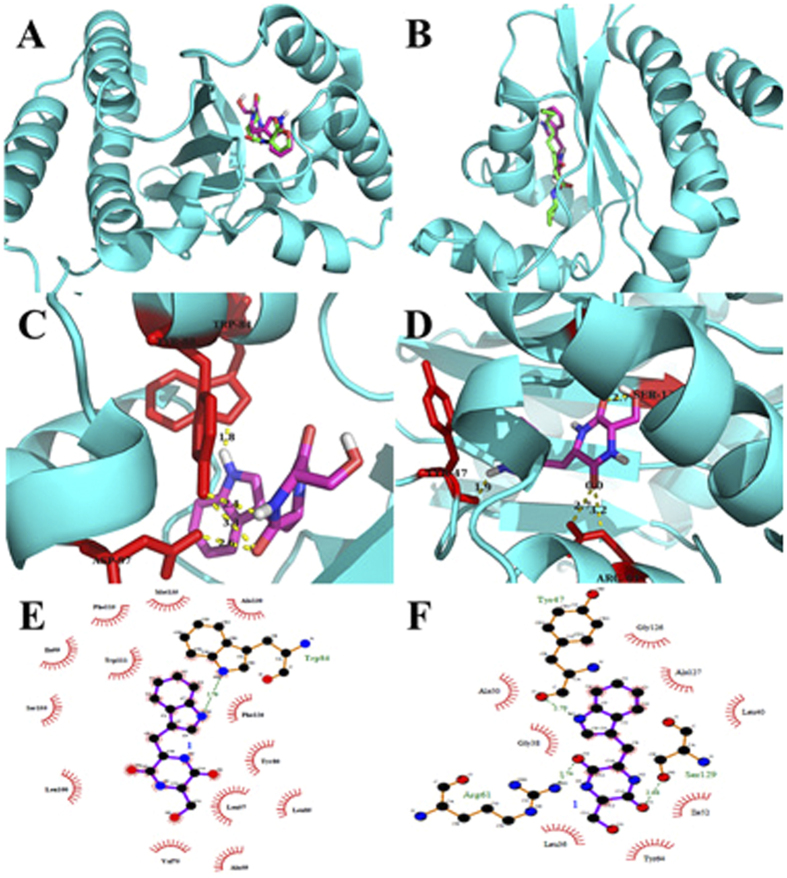
Docked conformation of natural ligand C_6_HSL (left) and 3-oxo-C_12_HSL (right) with green sticks and cyclo(Trp-Ser) with purple sticks into the active site of 3QP1 and 2UV0 receptor protein respectively (**A**,**B**); Analysis of docked cyclo(Trp-Ser) bound to 3QP1 (left) and 2UV0 (right) showing the key interactions in the binding pocket. The hydrogen bonds are shown with dotted yellow (**C,D**); Ligplot of cyclo(Trp-Ser) bound to 3QP1 (left) and 2UV0 (right) showing the key hydrophobic interactions (**E,F**).

The docking results with receptor CviR suggested that the compound cyclo(Trp-Ser) binds better, with docking energy −8.68 kcal/mol, than the natural ligand C_6_HSL (docking energy = −6.86 kcal/mol, Table 1). As shown in Fig. 4A, C_6_HSL and cyclo(Trp-Ser) reside at the same receptor pocket of CviR. The natural ligand C_6_HSL was found to form H-bonds with Asp97, Trp84 and Ser155, all of which were reported in the literature based on X-ray structural observations^46^,^48^. The docking results for cyclo(Trp-Ser) suggest that 3 H-bonds and 13 hydrophobic interactions with adjacent residues play key roles in successful docking (Fig. 4C,E). These interactions play an important role in the binding of CviR. Similar to the natural ligand, the cyclo(Trp-Ser) compound putatively forms H-bonds with Trp84 and Asp97. In addition, Tyr88 also acts as a key residue. Key hydrophobic contacts were observed between cyclo(Trp-Ser) and Trp111, Phe126, Tyr80, Leu57, Val75, Leu100, and Phe115.

The docking results using LasR showed that the docking energy of the natural ligand (−9.38 kcal/mol) is lower than the inhibitor (−8.40 kcal/mol) as shown in the Table 2. The natural ligand (3-oxo-C_12_HSL) was found to form H-bonds with Trp60, Ser129, Tyr56, and Asp73, while Arg61, Tyr47, and Ser129 were shown to be key residues when docking with cyclo(Trp-Ser). Only one same residue was found to interact with both ligand and cyclo(Trp-Ser). Besides, hydrophobic contacts between LasR and cyclo(Trp-Ser) include Leu40, Gly38, Ala127, Ile52, Gly126, Ala50, Leu36, Tyr64, Tyr56, and Asp73 as shown in Fig. 4D and F.

### Molecular dynamics simulation

**Figure 5:**
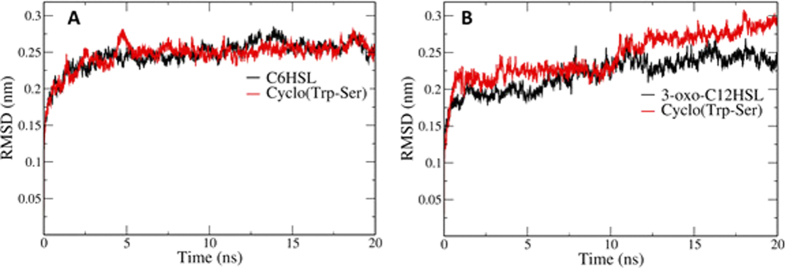
RMSD profile of simulated complexes. (**A**) Black color line represents CviR complex with signaling molecule, and red line represents CviR complex with cyclo(Trp-Ser); (**B**) Black color line represents LasR complex with signaling molecule, while red line represents LasR complex with cyclo(Trp-Ser).

Molecular dynamics simulations were performed to predict the conformation changes in the activation and deactivation of CviR and LasR receptors in the presence of the natural ligands and cyclo(Trp-Ser). The behavior of receptors throughout the simulations was screened based on the root-mean squared deviation (RMSD) profiles. As shown in Fig. 5A, the CviR-natural ligand is somewhat unstable compared to the CviR-cyclo(Trp-Ser) complex consistent with the higher docking energy. These results indicate that cyclo(Trp-Ser) is more easy to bind to CviR receptor than natural signaling molecule, and suggest that the inhibition of QS system in *C. violaceum* probably by competing with the natural ligand for binding to the same pocket. On the contrary, as shown in Fig. 5B, compared to the LasR-cyclo(Trp-Ser) complex, the LasR-natural ligand is more stable, and this also explains why the LasR receptor prefer binding with 3-oxo-C_12_HSL to cyclo(Trp-Ser).

## Discussion

QS in bacterial communication is crucial for survival and pathogenicity. The data presented here indicate that cyclo(Trp-Ser), previously isolated from the marine bacteria *Roseobacter* sp. and marine fungi (*Oidiodendron truncatum* and *Acrostalagmus luteoalbus)*, can be used as a QS inhibitor in *C. violaceum* and *P. aeruginosa*. It was evaluated for cytotoxic activities against nine cancer cell lines and exhibited weak activity with IC_50_ more than 10 *μ*M^49^,^50^. This report describes the first isolation of cyclo(Trp-Ser) from marine-derived *Rheinheimera aquimaris.* Additionally, it demonstrates the potential of cyclo(Trp-Ser) to inhibit QS systems in *C. violaceum* and *P. aeruginosa* for the first time. In the violacein assay, cyclo(Trp-Ser) effectively inhibited violacein production in the sub-MIC range of 0.05–0.2 mg/ml in the presence of the exogenetic natural signal molecule C_6_HSL. Cyclo(Trp-Ser) inhibits the QS system of *C. violaceum* not by the production of AHLs but perhaps by degrading AHLs or by interfering with the receptors. *P. aeruginosa* is an opportunistic human pathogen responsible for many life-threatening infectious diseases^51^,^52^,^53^, including chronic wounds, urinary tract infections, and respiratory tract infections, notably causing cystic fibrosis in hospitalized patients. Virulence factors and biofilm formation play important roles in its survival and invasion. The main virulence factors secreted by *P. aeruginosa* include pyocyanin, elastase and protease. To date, few DKPs have been reported to inhibit virulence factors except for cyclo(Gly-Gly), which induced significant reductions of pyocyanin, elastase and protease by 85%, 48% and 69%^54^, respectively. In this study, cyclo(Trp-Ser) demonstrated the ability to simultaneously inhibit the production of pyocyanin and elastase by 65% and 40%, respectively. Crystal violet biofilm, optical microscopy and SEM assays showed that 0.2 mg/ml cyclo(Trp-Ser) induced biofilm inhibition similar to that of the positive control AZM, which is accepted as a biofilm inhibitor and has been used as such in clinical treatment^45^,^55^. DKPs have been reported as antagonists against bioluminescence and swarming behavior. DKPs of cyclo(Ala-Val), cyclo(Pro-Tyr) and cyclo(Phe-Pro) produced by *P. aeruginosa* were identified as antagonists against 3-oxo-C6HSL-mediated bioluminescence induction. These results suggest that the DKPs may compete for the same LuxR binding site^56^. Cyclo(Pro-Tyr) isolated from *Alternaria alternata* inhibited the swarming behavior of a *Serratia liquefaciens N*-butanoylhomoserine lactone-dependent swarming motility mutant when combined with C_4_HSL^6^.

Molecular docking and molecular dynamics simulations studies were performed to determine the hotspot residues that interact with signaling molecules or inhibitors to change the conformation of the receptor for functional activity. *C. violaceum* uses the lux system (CviR/I proteins), which uses AHLs to regulate the production of violacein. At least three QS signaling systems have been reported in *P. aeruginosa.* The Las and rhl systems use the 3-oxo-C_12_HSL and C_4_HSL AHLs, while the other QS system uses 2-heptyl-3-hydroxy-4-quinolone as a signaling molecule. The Las system lies at the top of this signaling pathway and modulates both the rhl and the quinolone signaling systems. The docking results demonstrate that cyclo(Trp-Ser) inhibits the QS system of CviR in *C. violaceum* by competing with the natural ligand for binding to the same pocket with lower docking energy. And the RMSD profile also confirmed that the CviR-natural ligand is unstable compared to the CviR-cyclo(Trp-Ser) complex. On the contrast, docking results and RMSD profile confirmed that, compared to the LasR-cyclo(Trp-Ser) complex, the LasR-natural ligand is more stable. The former research reported that the absence of the long acyl chain in algal furanone led to the inability to H-bond with Asp73 and Thr75, and prevented the correct formation of LasR, which resulted in an unstable protein and the QS inhibition activity^47^. Therefore, these results suggested that the inhibition effect of cyclo(Trp-Ser) on QS in *P. aeruginosa* was probably due to the lacking of long side chain. However, the precise mode of interaction underlying these effects should be further elucidated by molecular insights based on the structure of the receptors bound to its inhibitors. Molecular docking methods and dynamics simulations have previously been combined to analyze the QS-inhibiting efficiency of screened compounds. For example, aspirin was shown to act as an anti-QS agent against *P. aeruginosa* by molecular docking studies^57^, and molecular docking and dynamics simulation studies were used to show that quercetin and cyanidin can act as competitive inhibitors for signaling molecules in the LasR receptor pathway^58^,^59^.

This study demonstrated that cyclo(Trp-Ser) not only efficiently inhibited violacein production in *C. violaceum* CV026 but also suppressed biofilm formation and other QS-regulated phenotypes, including pyocyanin production and elastase reduction, in *P. aeruginosa* PA01. *In silico* analysis demonstrated that in CviR-mediated QS system, cyclo(Trp-Ser) binds to the same pocket, albeit with different hydrogen bonds and hydrophobic interactions, resulting in a change in active protein conformation; in LasR-mediated QS system, the cyclo(Trp-Ser) probably performs anti-QS activity by interfering in the stability of the LasR receptor. All the data suggest that cyclo(Trp-Ser) may acts as a potential inhibitor of QS system both in *C. violaceum* CV026 and *P. aeruginosa* PA01. Further study on animals is recommended to demonstrate the QS inhibitory activity of cyclo(Trp-Ser) on pathogenic infectious.

## Materials and Methods

### Bacterial strains and culture conditions

*S. aureus* (ATCC25923), *E. coli* (DH5α), *C. violaceum* (ATCC12472 and CV026), *P. aeruginosa* PA01 (ATCC27853) and *C. albicans* (ID00147) were used in this study. Each isolate was cultured for 12 h with Luria Bertani (LB) broth containing 0.5% w/v yeast extract, 1% w/v tryptophan, 1.0% agar, and 0.5% w/v NaCl per 100 ml distilled water. The antimicrobial test used Mueller-Hinton broth (MHB) medium, containing 0.2% beef extract powder, 0.15% soluble starch, and 1.75% acid-hydrolyzed casein at pH 7.4.

### Fermentation and extraction of *Rheinheimera aquimaris* QSI02

The strain *R. aquimaris* QSI02 was obtained from marine sediment surrounding the Yellow Sea collected in the city of Qingdao (China). The fermentation was prepared in an Erlenmeyer flask (1000 ml) with 300 ml of defined medium composed of 5 g peptone, 2 g yeast extract and 0.1 g FePO_4_ dissolved in 1 L of seawater. The flasks were incubated on a rotary shaker at 28 °C at 160 rpm. After 48 h cultivation, 10 L of fermentation broth was filtered through cheesecloth to separate the broth supernatant and cells. The former was extracted by EtOAc and concentrated *in vacuo* to obtain 13.8 g of a crude gum.

### Isolation and structural elucidation

The crude extract was applied to a silica gel column and separated into four fractions (fractions 1–4) by eluting with a petroleum ether/MeOH gradient. Each fraction was tested by violacein assay to identify the target fraction, fraction 2. Fraction 2 was purified by Sephadex LH-20 (CHCl_3_/MeOH), and then semi-preparative HPLC was performed using a C18 column to obtain one compound, (10 mg, >95% purity by HPLC), identified as cyclo(Trp-Ser).

Cyclo(Trp-Ser) was obtained as a white, amorphous powder; (+)-ESIMS *m*/*z* 274.1 [M+H]^+^; ^1^H-NMR (600 MHz, CDCl_3_, TMS, *δ*) *δ*_H_: 10.88 (br s, 1 H, 1-NH), 7.88 (d, 2 H, 9′-NH, 12′-NH), 7.53 (d, 1 H, H-4), 7.32 (d, 1 H, H-7), 7.11 (d, 1 H, H-2), 7.04 (m, 1 H, H-6), 6.95 (m, 1 H, H-5), 4.90 (m, 1 H, 15′-OH), 4.00 (s, 1 H, H-8′), 3.67 (s, 1 H, H-11′), 3.29–3.33 (m, 1 H, H-15′), 3.16–3.18 (m, 2 H, 14′-CH_2_), 3.03–3.05 (m, 1 H, H-15′); ^13^C-NMR (150 MHz, CDCl_3_, TMS, *δ*) *δ*_c_: 173.58, 174.51 (C=O, C-10, C-13), 136.32(C-7a), 127.04 (C-3a), 127.45 (C-2), 122.31 (C-3), 118.44 (C-4), 119.46 (C-5), 111.96 (C-7),122.17 (C-6), 72.05 (C-11), 55.04 (C-8), 62.49 (C-15), 26.37 (C-14). This compound exhibited ^1^H and ^13^C NMR data consistent with literature values^41^,^49^.

### Determination of MIC

The MIC of cyclo(Trp-Ser) against selected bacteria was determined by a modified broth microdilution method according to the Clinical and Laboratory Standards Institute (CLSI, 2012). In brief, an overnight culture of selected microorganisms were diluted to an OD_600_ of 0.1 in MHB, and a 100-ml sample of the resulting microorganism suspension was mixed with an equal volume of a solution containing serial twofold dilutions (32–0.125 mg/ml) of cyclo(Trp-Ser) in a 96-well plate. The cultures were incubated for 24 h at 37 °C. The MIC of the compound was taken as the lowest concentration of cyclo(Trp-Ser) that inhibited visible bacterial growth. The use of a sub-MIC concentration was selected for the QS inhibition assays.

### Violacein assay

The effect of EtOAc extract, separated fractions and cyclo(Trp-Ser) on the production of violacein was quantified as previously described^35^,^60^. In summary, 10 ml LB broth containing different components were incubated with 200 *μ*l *C. violaceum* CV026 and 50 *μ*l C_6_HSL. Following incubation at 28 °C with shaking overnight, 1 ml of culture of each flask was centrifuged at 8000 × *g* for 10 min to obtain the insoluble violacein. The culture supernatant was discarded, and 1 ml DMSO was added to the tube. The solution was vortexed vigorously for 1 min and centrifuged at 8000 × *g* for 10 min to remove the cells. The absorbance was read with a microplate reader (M2^e^; Molecular Devices, Sunnyvale, CA, USA) at a wavelength of 585 nm. The harvested bacterial cells were re-suspended in 1 ml sterile water to evaluate cell growth by measuring the optical density at 600 nm.

### Biofilm assay

The biofilm assay was conducted in 96-well polystyrene plates as previously described, with minor modifications^61^. *P. aeruginosa* PA01 was inoculated into LB medium at an initial OD_600_ of 0.05 and cultured with cyclo(Trp-Ser), DMSO or AZM at 37 °C for 24 h. The broth was discarded by washing with PBS three times. The plates were dried at ambient temperature for 20 min and then stained with 1% crystal violet for 5 min. The stained biofilms were washed with PBS, followed by the addition of 200 *μ*l 95% ethanol. The absorbance was read at a wavelength of 545 nm.

The effects of cyclo(Trp-Ser) on biofilm formation were further confirmed with microscopy (B204LED, Optec, China) and SEM^62^ (S-4800, Hitachi, Japan). In brief, 3 ml *P. aeruginosa* PA01 (OD_600_ = 0.05) were cultured with cyclo(Trp-Ser), DMSO or azithromycin in a 6-well plate. Sterile slide/cover glasses were placed in the bottom of the wells and incubated at 37 °C for 3 days. After incubation, the non-adherent cells were removed by washing with PBS 3 times. The cover glasses were used for optical microscopy after silver staining and the slide glasses for scanning electron microscopy after being fixed with 2.5% glutaraldehyde and coated with gold.

### Pyocyanin assay

The pyocyanin assay was performed in a quantitative chemical method as previously described^60^,^63^. *P. aeruginosa* PA01 was inoculated into LB medium at an initial OD_600_ of 0.05 and was cultured with cyclo(Trp-Ser), DMSO or azithromycin at 37 °C for 18 h. The broth (5 ml) was extracted using CHCl_3_ (3 ml), and the organic layer was mixed with 0.2 M HCl. The water layer was collected by centrifugation, transferred to a 96-well microplate in triplicate, and measured at 520 nm. The percent change in absorbance was then calculated to determine the decrease in pyocyanin.

### Elastase activity assay

Elastase activity was determined using a modification of a previously reported method^60^,^64^. *P. aeruginosa* PA01 was cultured to an initial OD_600_ of 0.05 for 24 h in LB media containing cyclo(Trp-Ser), DMSO or AZM. The top layer (100 *μ*l) was collected by filtration using a 0.22 *μ*m nylon filter, and then 10 mg elastin-congo red (ECR) and 900 *μ*l buffer (100 mM Tris-HCl/1 mM CaCl_2_, pH 7.2) were added. After incubation at 37 °C for 3 h, the samples were centrifuged to remove insoluble ECR. Both the control and samples were tested using OD_495_ nm. The percent change in absorbance was then calculated to determine the elastase activity.

### Docking studies

Molecular docking studies were performed to identify the conformational changes in the protein structure due to the interactions of cyclo(Trp-Ser) with the CviR and LasR receptors. All computations were carried out by a series of software programs: ChemBioOffice 2010, Autodock 4.2.6^65^, LigPlot^+^ v.1.4^66^ and PyMol 1.5.0.3^67^. The natural ligands used as controls and the novel molecule, cyclo(Trp-Ser), were first drawn by ChemBioOffice software, and the energies were minimized with ChemBio3D Ultra 12.0. The crystal structures of *C. violaceum* CviR (PDB ID: 3QP1, resolution 2.0 Å) and *P. aeruginosa* LasR (PDB ID: 2UV0, resolution 1.8 Å) were downloaded from the protein data bank (PDB) and used as target receptor proteins^46^,^58^. The PDB 2UV0 structure contains four chains (E, F, G and H) whose conformation is similar. The H chain is the longest and contains the preferred binding site for the natural ligand. First, the water molecules, co-crystallized ligands and other chains of 2UV0 were eliminated. The proteins were then modified by adding polar hydrogens, and the Gasteiger charges were computed. The grid box was set to the whole receptor involved in the active site, and the docking parameter files were created using the Lamarckian genetic algorithm (GA) before the program was run. The number of GA runs was set to 100 replications; the other settings were set to their default values. The results of the docking computations were ranked by binding energy. The PyMol molecular graphic system and LigPlot were used to visualize the conformations and interactions between the ligands and the target proteins.

### Molecular dynamics simulation

Molecular dynamics simulation studies were performed using the GROMACS 4.5.2 package^68^ to confirm the binding mode and to investigate the dynamic behavior following the molecular docking. The Gromos43al force field was used, and the conformation with lowest docking energy was selected for molecular dynamics simulation. A cubic box with a side length of 1.2 Å was installed along with the SPCE water model as a solvent. The system was equilibrated well before final simulation of 20 ns with a time step of 2 ps.

## Additional Information

**How to cite this article**: Sun, S. *et al*. A diketopiperazine factor from *Rheinheimera aquimaris* QSI02 exhibits anti-quorum sensing activity. *Sci. Rep.* **6**, 39637; doi: 10.1038/srep39637 (2016).

**Publisher's note:** Springer Nature remains neutral with regard to jurisdictional claims in published maps and institutional affiliations.

## Supplementary Material

Supplementary Information

## Figures and Tables

**Table 1:** Details of docked complex of CviR receptor protein with natural ligand and compound cyclo(Trp-Ser).

Molecules	Binding Energy (kcal/mol)	Hydrogen bonding interactions	Key Hydrophobic interactions
Natural ligind (C_6_HSL)	−6.86	Asp97, Trp84, Tyr80, Ser155	Leu100, Leu85, Ile99, Tyr88, Trp111, Phe126, Trp111, Phe126, Tyr80
Cyclo(Trp-Ser)	−8.68	Asp97, Trp84, Tyr88	Leu57, Val75, Leu100, Phe115

**Table 2:** Details of docked complex of LasR receptor protein with natural ligand and compound cyclo(Trp-Ser).

Molecules	Binding Energy (kcal/mol)	Hydrogen bonding interactions	Key Hydrophobic interactions
Natural ligind (3-oxo-C_12_HSL)	−9.38	Trp60, Ser129, Tyr56, Asp73	Phe101, Leu110, Ala105, Trp88, Tyr93, Gly126, Ala50, Tyr47, Tyr64, Thr75
Cyclo(Trp-Ser)	−8.40	Arg61, Tyr47, Ser129	Leu40, Gly38, Ala127, Ile52, Gly126, Ala50, Leu36, Tyr64
