# Supplementary material for: A diketopiperazine factor from Rheinheimera aquimaris QSI02 exhibits anti-quorum sensing activity
Source: Sci Rep. 2016 Dec 21;6:39637. doi: 10.1038/srep39637 (PMC5175134; doi:10.1038/srep39637)
Supplement: Supplementary Information [file srep39637-s1.pdf]

Supplementary Information

A diketopiperazine factor from *Rheinheimera aquimaris* QSI02 exhibits anti-quorum sensing activity

Shiwei Sun, Xiaoyun Dai, Jiao Sun, Xiangguo Bu, Caihong Weng, Hui Li and Hu Zhu*

Supplementary Figure 1: The ^{1}H-NMR spectrum of compound cyclo(Trp-Ser) in $CDCl_3$.

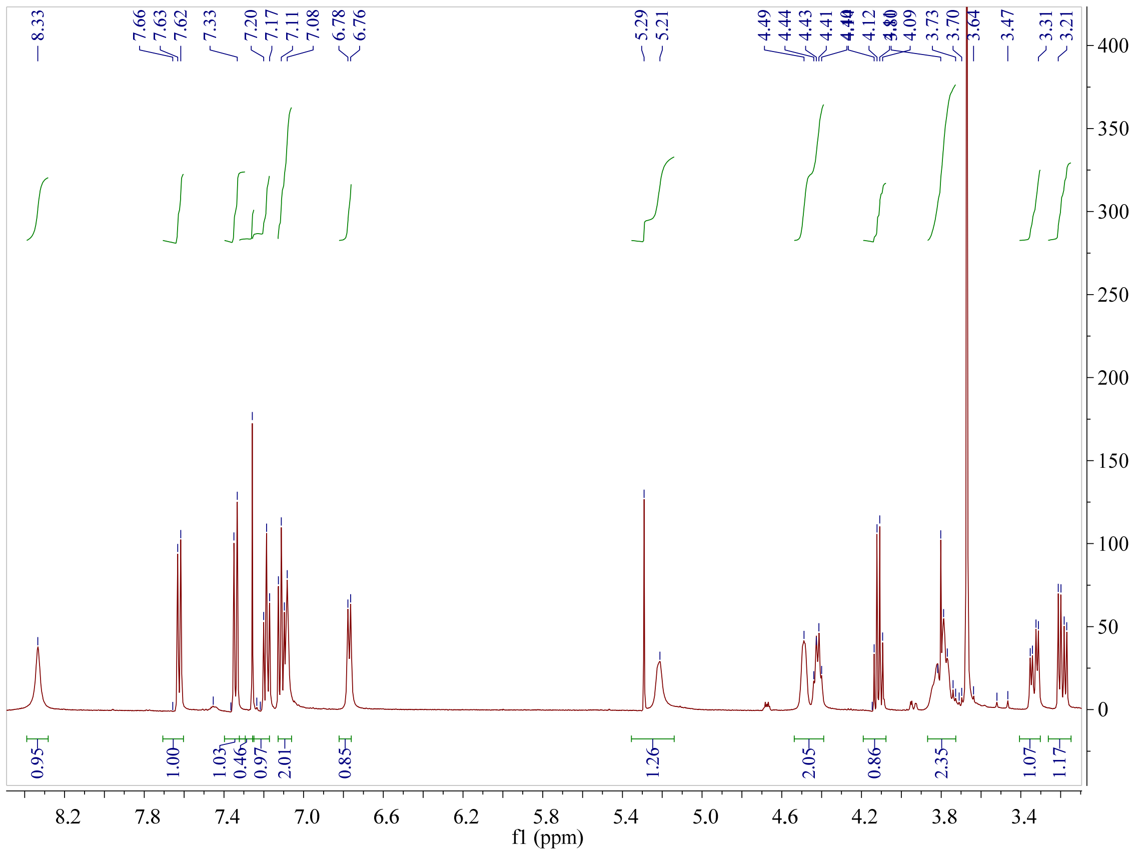

Supplementary Figure 2: The ^{13}C-NMR spectrum of compound cyclo(Trp-Ser) in $CDCl_3$.

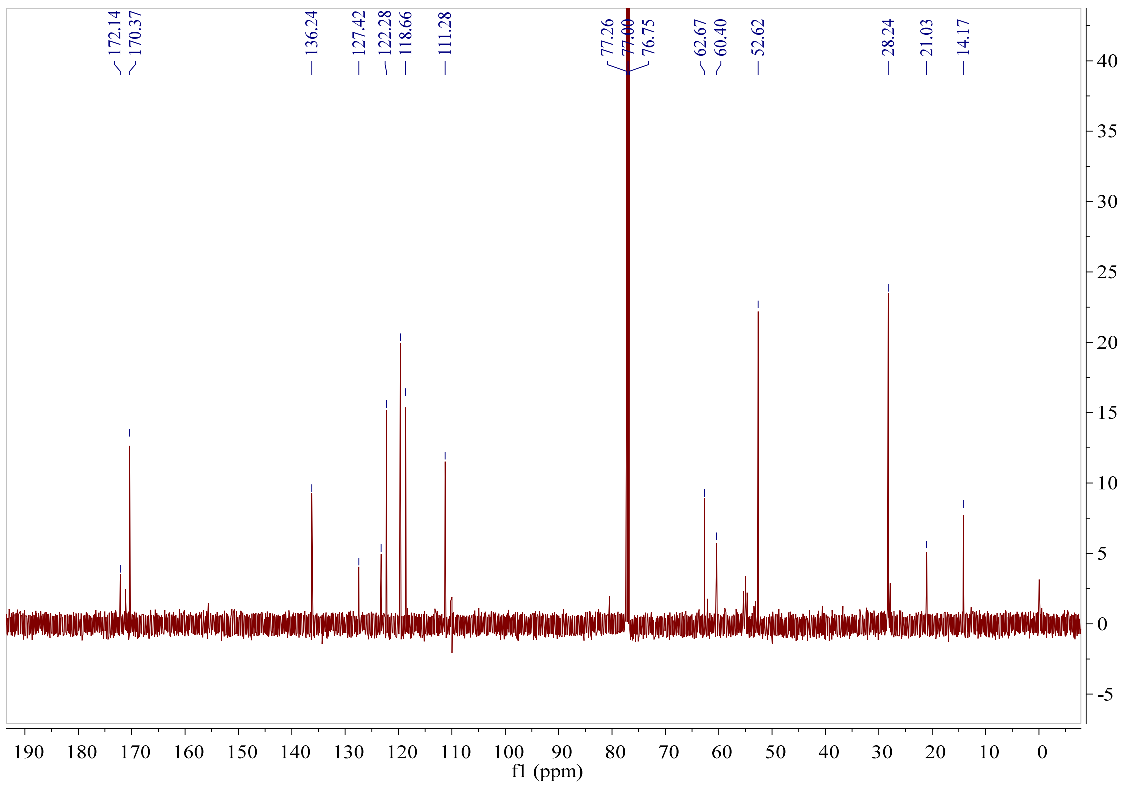

Supplementary Figure 3: The MS spectrum of compound cyclo(Trp-Ser).

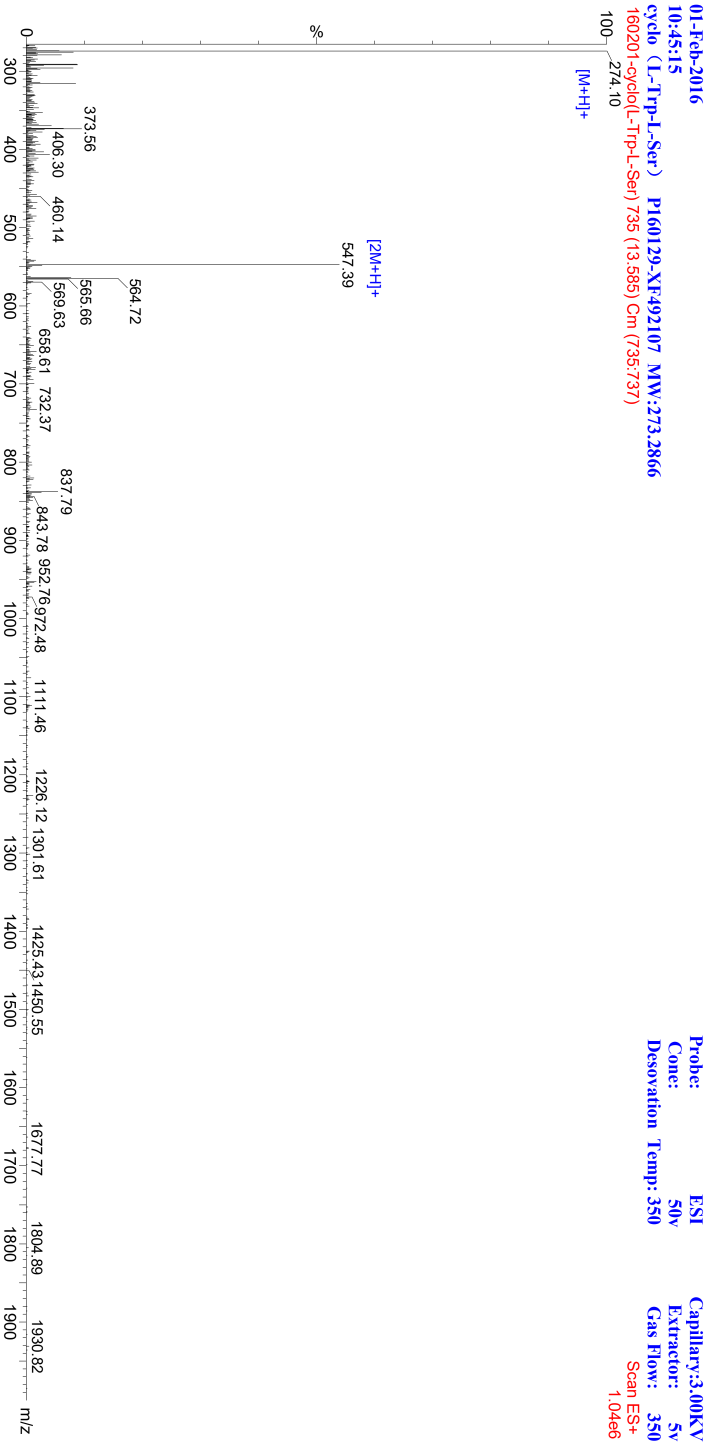

Supplementary Figure 4: (A) Overlay of docked pose of C_6HSL (native ligand; in green sticks) with its X-ray structure (in yellow sticks) for 3QP1 protein. (B) Analysis of docked C_6HSL bound to 3QP1 showing the key interactions in the binding pocket.

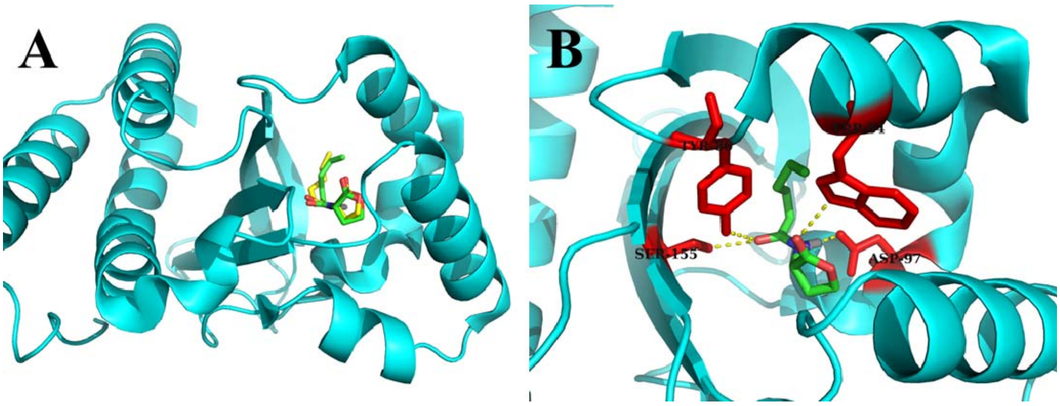

Supplementary Figure 5: (A) Overlay of docked pose of 3-oxo-C_{12}HSL (native ligand; in green sticks) with its X-ray structure (in yellow sticks) for 2UV0 protein, with RMSD=1.56 Å. (B) analysis of docked 3-oxo-C_{12}HSL bound to 2UV0 showing the key interactions in the binding pocket.

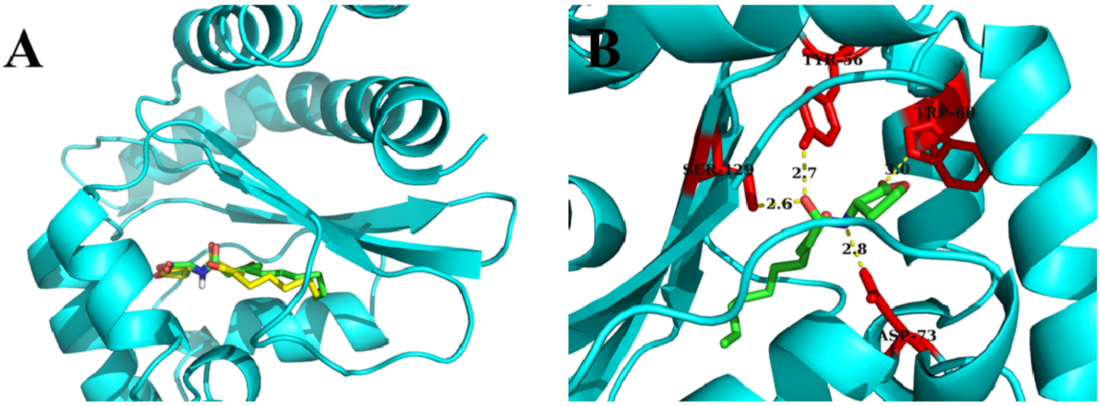

Supplementary Table 1 MICs values of cyclo(Trp-Ser) and azithromycin against different microorganisms.

Microorganisms	MIC(mg/ml)	
	cyclo(Trp-Ser)	azithromycin
Staphylococcus aureus ATCC25923	3.2	0.2
Escherichia coli	6.4	0.2
Chromobacterium violaceum	3.2	0.4
Chromobacterium violaceum CV026	3.2	0.4
Pseudomonas aeruginosa PA01	6.4	0.2
Candida albicans 00147	6.4	0.8
